# Ultraprecise Controller for Piezoelectric Actuators Based on Deep Learning and Model Predictive Control

**DOI:** 10.3390/s23031690

**Published:** 2023-02-03

**Authors:** Jokin Uralde, Eneko Artetxe, Oscar Barambones, Isidro Calvo, Pablo Fernández-Bustamante, Imanol Martin

**Affiliations:** 1 Department of Systems Engineering and Automatic Control, Faculty of Engineering of Vitoria-Gasteiz, University of the Basque Country (UPV/EHU), 01006 Vitoria-Gasteiz, Spain; 2 Department of Electrical Engineering, Faculty of Engineering of Vitoria-Gasteiz, University of the Basque Country (UPV/EHU), 01006 Vitoria-Gasteiz, Spain

**Keywords:** piezoelectric actuators, hysteresis, control systems, neural networks, model predictive controller (MPC)

## Abstract

Piezoelectric actuators (PEA) are high-precision devices used in applications requiring micrometric displacements. However, PEAs present non-linearity phenomena that introduce drawbacks at high precision applications. One of these phenomena is hysteresis, which considerably reduces their performance. The introduction of appropriate control strategies may improve the accuracy of the PEAs. This paper presents a high precision control scheme to be used at PEAs based on the model-based predictive control (MPC) scheme. In this work, the model used to feed the MPC controller has been achieved by means of artificial neural networks (ANN). This approach simplifies the obtaining of the model, since the achievement of a precise mathematical model that reproduces the dynamics of the PEA is a complex task. The presented approach has been embedded over the dSPACE control platform and has been tested over a commercial PEA, supplied by Thorlabs, conducting experiments to demonstrate improvements of the MPC. In addition, the results of the MPC controller have been compared with a proportional-integral-derivative (PID) controller. The experimental results show that the MPC control strategy achieves higher accuracy at high precision PEA applications such as tracking periodic reference signals and sudden reference change.

## 1. Introduction

Piezoelectric actuators (PEA) are positioning systems that achieve a mechanical displacement from an applied voltage. They have been widely used in the field of micro- and nano-positioning applications due to their high displacement resolution [1] (around one micrometer) and high actuation force [2] (usually in the range of several hundred newtons). In addition, these systems offer not only high-speed response, but also show high stiffness. A further advantage is their size, which is an asset due to the downsizing trend in actuators today [3].

Due to these benefits, PEAs may be used at a vast number of applications such as energy recovery [4], computer components [5], motor design [6], machine tools [7], and micro-drones [8]. Furthermore, in the last few years, PEAs have been the subject of study for several medical uses, such as needle positioning for complex injections [9], micro grippers [10], and drug delivery systems [11].

Regardless the benefits of PEAs, the performance of these actuators downgrades due to undesirable non linear effects that appear in piezoelectric materials such as vibration dynamics [12], creep [13], and hysteresis. Vibration dynamics, on the one hand, are caused by the input voltage excitation that operates the equivalent mechanical system, although this should be considered when the input frequency reaches the resonance of the PEA [14]. Creep, on the other hand, is produced by the polarization that remains in time during the actuation in a quasi-static situation [15]. Finally, hysteresis is provoked by the non-linear nature of the piezoelectric effect, which combines mechanical actions and electric fields [16]: The material poles have arbitrary orientations that align when a voltage is applied, but the release of this action gives way to a different direction [17]. Therefore, it is known to be a memory effect, since it depends on the previous history of the piezoelectrical material [18].

Hysteresis is one of the most important and studied effects in PEAs, since the error can reach up to 22% [19], which is a significant value when high precision is required in guidance systems. Furthermore, this phenomenon cannot be disregarded since it not only affects a desired position but also it can yield the system to the instability [20].

As above-mentioned, piezoelectric actuators need to overcome the impacts of nonlinear hysteresis to achieve high-precision positioning, but they also need to cope with the uncertainties of the model. For that reason, it is necessary to implement appropriate compensation designs that minimize the nonlinear hysteresis [21].

The design of models that reproduce the nonlinear characteristics of PEAs is the basis of improving the behavior using feedforwarding control techniques. Several modeling methods have been proposed for this purpose. In the case of hysteresis, modeling has been done using two alternative methods: rate-independent and rate-dependent. The only factor affecting the model output in the rate-independent method is the amplitude of the input voltage. The major benefit of these models is that they are easy to identify and simple to implement [22,23]. Within this method, three different model theories can be distinguished: (1) the theory of dynamic modeling, which is represented mathematically by a collection of differential equations. Some model examples include the Jiles–Atherton model [24], the Duhem model [25], the Bouc–Wen model [26], the Backlash-Like model [27], the Maxwell model [28], and the approximated Polynomial model [29]; (2) the operator modeling theory, including Prandtl–Ishlinskii (PI) [30], Krasnoselskii–Pokrovskii (KP) [31], and Preisach [32] models; (3) the intelligent modeling theory. This approach models systems with hysteresis based on the concept of the computational intelligence [33]. This kind of models are based entirely on data-driven methodologies and have rather simple shapes. Fuzzy models [34], autoregressive-moving-average (ARMA) [35], machine learning based models [36], and neural network-based models [37,38] are a few examples. The latter type of modeling will be the one implemented in this research.

These models provide good approximations for hysteresis when the frequency of the input signals is low. However, when the frequency rises, it becomes increasingly clear how the model differs from the actual behavior of the PEA. This happens because these models do not consider the rate-dependent behavior of the hysteresis. As a consequence, the performance of PEAs in open-loop systems can be significantly reduced, and they may even push the closed-loop system toward instability. In fact, some research works show the strong dependency between the hystereis behavior of the PEAs against the input rate [39]. Moreover, the first two ways for modeling PEAs typically use complex structures, which require high computing costs, and require the incorporation of additional creep and dynamic models [40].The second class of hysteresis models, the so-called rate-dependent models, have been used to investigate this type of behavior. The output of these models depends on the frequency and amplitude of the input signal. Models such as the rate-dependent PI [41], rate-dependent Preisach model, and modified rate-dependent PI (MPI) are examples of this class [42]. Despite the fact that these models accurately depict hysteresis behavior, they are frequently complex. This makes it challenging to identify model parameters and design controllers capable of compensating the hysteresis [22]. However, rate-dependent intelligent modeling has also been developed using machine learning [36], neural networks [43], or fuzzy systems [44]. A new approach for modeling the hysteresis of the PEAs has been made possible by means of these intelligent approaches, which have demonstrated significant gains in terms of modeling accuracy.

Numerous control methods are offered for PEAs positioning based on the use of models. Frequently, they are used as inversion-based models. Thus, an inverse model for the hysteresis may be used in order to achieve a feedforward controller to compensate this phenomenon. Feedback techniques are then employed to cope with its dynamics, external disturbances, as well as other minor nonlinearities. For example, in reference [45], the authors created a PID controller with a feedforward compensator, based on the numerical inverse Preisach model, achieving a maximum error of 20 μm. Other researchers [46] used the Bouc–Wen and the Dahl model as feedforward with a PI controller, achieving a RMS value of 0.157 μm and 0.146 μm, respectively.

A non-linear method called sliding mode control (SMC) uses a discontinuity to drive the system over a sliding surface [47]. The key benefit of this approach is the ability to withstand uncertainty and outside shocks. The authors of Ref. [48] applied the traditional SMC in PEAs for force control obtaining appropriate results when sine signals were used as references. In a study by Chouza et al. [49], a similar methodology was used to build an SMC-based PID surface in a commercial PEA. In this work, several reference signals were tested, including the ramp, constants values, and sine wave signals. The sine waves indicated an inaccuracy of about 5%, despite advances in the reduction of the error when the ramp and constant references were used. However, it was determined from the background analysis that the chattering caused by the discontinuous characteristic is the primary drawback of the SMC technique. This is an unintended consequence since it causes the actuator to wear down faster and lose more energy [50].

Other works analyze the implementation of different control schemes. For example, the researchers in Ref. [51] make use of robust control techniques alongside fuzzy logic to learn the models of the actuators and mitigate the hysteresis. Their approach achieves an accuracy with a margin of error of 0.18 μm. The authors of Ref. [21] proposed a hybrid nonlinear robust control design that integrates a feedback linearization control method and a robust compensator, also achieving a 1 μm accuracy.

However, in the case of high-speed and high-frequency motion, conventional controllers are unable to meet the requirements for high-precision control, as they suffer from the limited bandwidth due to the presence of highly resonant frequencies. Modern intelligent control techniques have therefore drawn attention due to their improved performance. In particular, the Model predictive control (MPC) scheme is an example of an intelligent technique widely used in practical applications that efficiently reduces disturbances and demonstrates the needed robustness [1]. Based on model input, a nonlinear model forecasts the future displacement of the PEA, and the actual control signal applied to the PEA is calculated in accordance with the expected displacement [37]. MPC often uses a series of expected future outputs over a specific prediction horizon for optimization, which necessitates running the model more than once, incrementing the computing load. Linearizing the prediction model is a common technique used to ease calculations [52]. The MPC has a reasonable performance on the tracking control for different frequency signals when neural networks [37] or fuzzy model [53] are used.

Based on the state of the art literature, this work presents how the hysteresis behavior of one PEA at a specific frequency has been modeled by means of an artificial neural network. A rate-independent model could be created, but this approach requires large amounts of data in order to train an optimal neural network over a wide bandwidth.

The required data for the ANN is extracted from a commercial PEA developed by Thorlabs. Then, based on that ANN model, a MPC controller is introduced because of its high performance with different nonlinear models. This approach was tested in real time over the same commercial PEA supplied by Thorlabs, obtaining a maximum error in the tracking of a triangular and sinusoidal signal of 0.05 μm, values lower than those obtained by the authors of Refs. [49,51]. The authors of Ref. [49] used a Sliding Mode-Based Robust Control and tried to track a sinusoidal reference wave, where a maximum error of 0.2 μm was achieved. This same maximum error was achieved by the authors of Ref. [51] using a robust fuzzy control scheme.

Other articles such as [37,40] show a control scheme similar to the one presented in this work but with significant differences. Both papers model the hysteresis of the PEA using a neural network and then make the feedback control using a MPC scheme.

The authors of [40] made use of a neural network to model the hysteresis of the PEA. However, their approach employed a second neural network for implementing an ANN based feed forward control aimed at improving the performance of the MPC. In this sense, the computational cost of this control scheme is considerably higher than the proposed control scheme. In addition, the tracking performance of this control scheme is comparable to our approach in percentage but not in range. Actually, authors in [40] report a tracking error below 4nm for a displacement of 3 µm (tracking error of 0.13 %), whereas in our approach the tracking error is below 50 nm for a much longer displacement, 38 µm (tracking error of 0.13%).

Moreover, the difference in the tracking error obtained at different frequencies in the reference signal is relatively smaller in our proposal, achieving a more robust approach under frequency variations.

In the case of paper [37], two neural networks were also used, one to model hysteresis and one to model the dynamics. In this case, the output of the hysteresis sub-model was an equivalent mechanical force that acted as input in the dynamic sub-model, whose output produced the actual displacement of the PEAs. The network that modeled the dynamics was trained at various frequencies such as 1, 10, 50, 100 and 200 Hz. Thus, the amount of data required for training the ANN (and therefore the computational cost of the algorithm) is larger than that used in our approach. This rate independent ANN, together with the fact of having two ANN, requires a higher computational cost, making more difficult its implementation over low-cost real time processors. Moreover, the sample time used for the real time implementation of this control scheme is 0.05 ms, which is a very small value for a high computational control schemes like the proposed one. The authors implement their algorithm over a computer using the Matlab Real-Time Windows Target. Unfortunately, they do specify the processor used, so it is not possible to compare with our real time implementation.

Obviously, considering the small sample time used (0.05 ms versus 1 ms), and the small displacement of the PEA (9 µm) the tracking results are better, but the computational cost may introduce severe difficulties at implementation time. In addition, in this paper the authors have only presented experiments with sinusoidal references, whereas in our work different types of references are presented, namely triangular, sinusoidal and step references. Using a unique type of reference makes more difficult to reach conclusions about the robustness of this control scheme under different types of references.

The structure of this paper is organized as follows: Section 2 provides an overview of the hardware that was used in the research, a brief description of the hysteresis, and an explanation about how the HW block works. Section 3 describes the artificial neural networks designed for modeling the hysteresis behavior regarding its architecture. Section 4 shows the two different controllers that have been implemented: a MPC (Section 4.1) and a PI (Section 4.2) are compared. Section 5 presents the results of both the training of the network in Section 5.1 and the results of the tracking of a reference signal using the two controllers mentioned above in Section 5.2.

## 2. Materials and Methods

### 2.1. Hardware Description

The main hardware use in the research was based on the Thorlabs (Newton, NJ, USA) PK4FYC2 PEA.This PEA is a stack actuator that has several piezoelectric chips stuck with epoxy and glass beads. Its dimension is 7.3 × 7.3 × 36 mm. The nominal maximum displacement is of 38.5 μm with a drive voltage between 0 and 150 V. The resonant frequency of the PEA is 34 kHz and the maximum error caused by the hysteresis according to the manufacturer is of 15%. To achieve better resolution in measuring the displacement, this device uses the resistance variation of four strain gauges, attached to it, arranged forming a Wheatstone bridge. The maximum blocking force that can support this PEA is of 1000 N.

The signal acquisition and generator controller used was a dSPACE board, specifically the DSP1104. This board allows inputs and outputs in the range of 0–10 V and is capable of running in real-time interface (RTI). The board was connected via PCI peripheral interface to a PC with an Intel 64 2.8 GHz microprocessor and 32 GB of memory. The PEA signals passed through a Thorlabs AMP002 pre-amplifier that gives a signal of 0–2 V. Next, a measurement cube reader Thorlabs KSG101 transformed this signal to equal the DSP board range of 0–10 V. Control Signal passed through the Thorlabs KPZ101 driver that transforms the controller 0–10 V signal to 0–150 V. Table 1 summarizes the technical details of the hardware used in the research.

**Figure 1:**
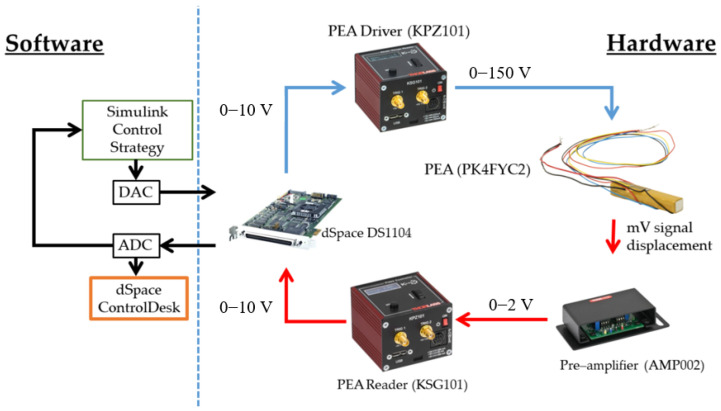
Hardware and software workflow.

The control structure was designed in Simulink by MathWorks and implemented into the embedded hardware through dSPACE real-time interface. In Simulink, the Deep Learning and Model Predictive Control Toolboxes were used to design the PEA model and the MPC controller, respectively. All the data acquisition, supervision, and tuning were done with the graphical user interface Control Desk, part of the dSPACE software package. Posterior data treatment and process was done with Matlab by MathWorks. The sample rate used was of 1 kHz since it suits the relationship between the hardware limitations and control needs. Figure 1 show a schematic description of the hardware and software workflow.

### 2.2. Hysteresis Description

Hysteresis phenomenon is the main reason of the non-linearities of PEAs. The hysteresis graph can be obtained using periodical signals such as sines and triangular wave signals and plotting the registered displacement versus the input voltage. In this study the triangular wave signal is used to obtain the hysteresis. The election of this signal is justified since a triangular wave signal is a more complex source due to the high harmonics that compose it and the sharp slope changes on the ends.

**Figure 2:**
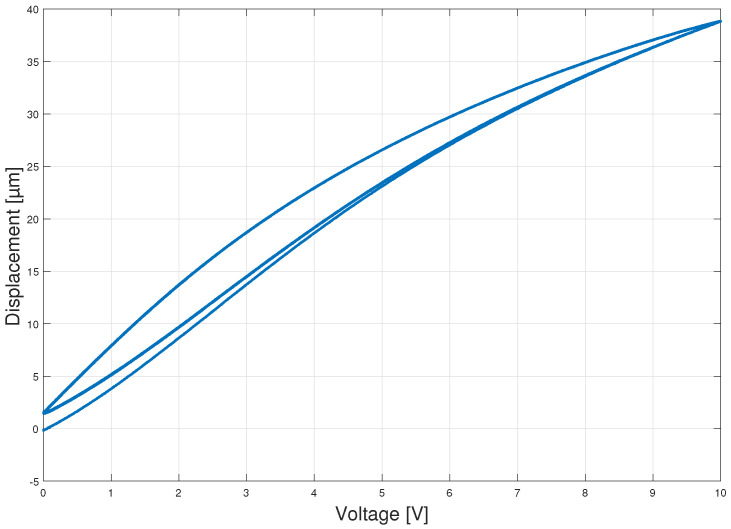
Hysteresis graph description of the commercial Piezoelectric Actuator (PEA).

Figure 2 shows the hysteresis that appears with periods of 10 s. The signal was referenced to zero before measuring with 0 V at the input. All the curves in the Figure show a strong non-linearity. The first ascent starts at zero, reaching the upper limit. This path is only traversed the first time, in subsequent cycles the paths converge to a hysteresis curve from the upper boundary to the lower convergence point. The lower convergence point is offset from the origin, so that after the first cycle it is impossible to reach displacements below this point. The asymmetry between ascent and descent makes it difficult to obtain an accurate mathematical model of the piezoelectric.

## 3. PEA System Modeling with ANN

In PEA, hysteresis and rate-dependent dynamic properties are the key nonlinear characteristics that need to be modeled. Hysteresis, as previously established, is a type of memory phenomenon that links the displacement of PEA to states and signals from the past. ANNs offer several benefits, such as self-learning and simplicity, and are ideal to train one hysteresis curve. Input, hidden, and output are the three mathematically related layers that must be included in an algorithm to be considered an ANN [54]. This biological idea is derived on brain neurons’ capacity to identify, pick up on, and modify behavior based on prior experiences (also known as neuroplasticity) [55]. Because of this, nonlinear dynamic systems can be approximated using these attributes based on the mathematical formulation [56]. Nevertheless, the rate-dependent dynamic is a difficult feature to implement in an ANN due to the large amount of data needed to know the different hysteresis formed over a large bandwidth. Therefore, the ANN will be subject to a single frequency and frequencies contiguous to this one, thus maintaining its simplicity and low computational cost.

**Figure 3:**
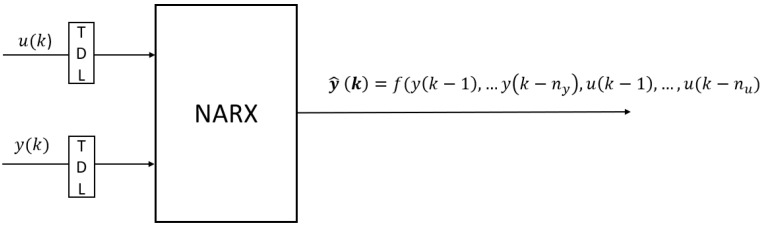
NARX series parallel type-model structure.

In this research, the ANN is a self created one that is used as an approximator for NARX representation. As will be discussed later, the network will provide the PEA displacement, which will be fed back to the network to know its direction of displacement. Therefore, the network needs previous states to conclude the next displacement. This fact can be described by the following Equation (1)

(1) $$\hat{y}\left(k\right)=f\left[y\left(k-1\right),y\left(k-2\right),\ldots ,y\left(k-n_{y}\right),u\left(k-1\right),u\left(k-2\right),\ldots ,u\left(k-n_{u}\right)\right]$$

where y(i) and u(i) indicate the displacement and control signal respectively of the PEA at *i* operating point. Integers $n_{y}$ and $n_{u}$ are the sizes of historical memory of y(i) and u(i), respectively. This indicates that we are dealing with a NARX model, where the next value of the dependent output signal is regressed on previous values of the output signal and previous values of an independent (exogenous) input signal as shown in Figure 3. It should be noted that the output of the NARX network it is considered to be an estimate of the output of the PEA dynamic system that we are trying to model. The output could be fed back to the input of the ANN as part of the parallel NARX architecture. Because the true output is available during the network training process, a series parallel architecture could be created using the true output rather than feeding back the estimated output, as shown in Figure 3, where Tapped Delay Line (TDL) blocks are used to represent the delayed signal value. This has two advantages: The input to the ANN is more precise and the resulting network has a purely feedforward architecture, so static back propagation can be used for training.

In order to improve the performance of MPC, a number of predicted outputs $\left[\hat{y}\left(k+1\right),\ldots ,\hat{y}\left(k+n_{p}\right)\right]$ inside the prediction horizon $n_{p}$ are required at instant *k*. Usually, the model successfully outputs them when past predictions are included in historical states, that means, using $\hat{y}\left(k+i\right)$ as y(k+i) when predicting $\hat{y}\left(k+i+1\right)$. This repetitive process is expensive computationally so the Equation (1) is changed to produce all $n_{p}$ future predictions at once as shown in the following Equation (2):

(2) $$\left[\hat{y}\left(k+1\right),\ldots ,\hat{y}\left(k+n_{p}\right)\right]=f\left[y\left(k\right),y\left(k-1\right),\ldots ,y\left(k-n_{y}\right),u\left(k+n_{p}-1\right),\ldots u\left(k\right),\ldots u\left(k-n_{u}\right)\right]$$

**Figure 4:**
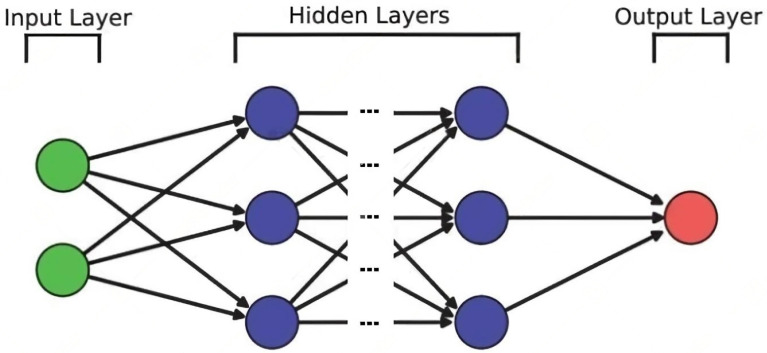
Standard ANN scheme with multiple hidden layers for two inputs and one output.

Here $[u\left(k+n_{p}-1\right),\ldots u\left(k\right)$ are the output signals of the controller. This model works well when the prediction horizon is small; otherwise, the predictive accuracy may decrease. Having considered the NARX model, the ANN that approximates it must be described. In this research, as mentioned above, the ANN and its architecture is a self created one and tries to model the PEA dynamic at 0.1 Hz. The architecture of the ANN consists of the linking of an input layer, three hidden layers, and an output layer as shown in Figure 4. The input layer is fed with two data: the voltage applied to the PEA and the derivative of the displacement signal that will indicate on which of the two hysteresis curves the displacement is currently located. The hidden layers, meanwhile, are made up of two fully connected layers and one Long Short-Term Memory (LSTM) layer. Based on the optimization procedure by means of simulations, the amounts of neurons for this layer were determined on a range from 90 to 120 for the LSTM layer and 195 to 220 for the fully connected layer. The optimal number of neurons for each layer are as follows: 100 for LSTM and 200 for the fully connected layer. Finally, for the output layer it we chose a regression output layer, from which the displacement is obtained.

## 4. Control Structure

In this research, two control architectures have been designed and implemented to test their efficiency and compare them afterwards. First, a basic PID control is implemented and tuned, trying to achieve the best possible tracking. The second control, on the other hand, is carried out by means of an MPC, based on the model created by ANN.

The two controllers are designed and tuned based on trial and error basis using the created ANN as a plant in MATLAB simulations. In this way, the obtained controllers can be extracted to do the actual control of the PEA and see their performance.

### 4.1. Model Predictive Control

Model predictive control (MPC) is an ideal control method that minimizes a cost function for a constrained dynamical system over a finite, prediction horizon.

**Figure 5:**
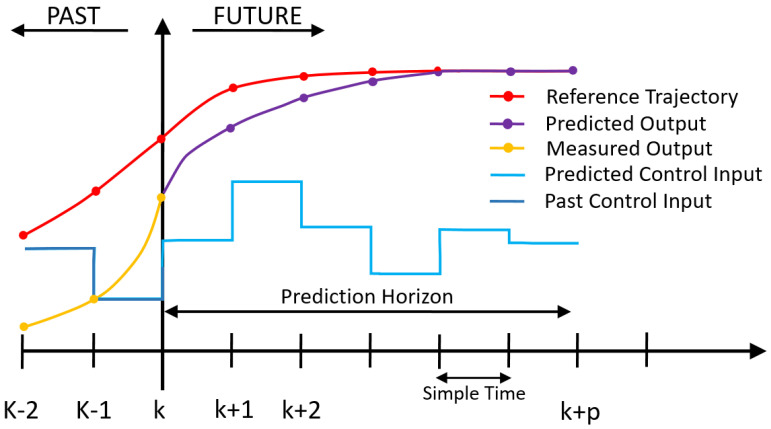
Discrete MPC operating scheme.

Model predictive controller uses a model of the plant to predict the evolution of the system over a finite time steps and solve an optimization problem to calculate the control vector that minimized a certain cost function as shown in Figure 5. The prediction horizon determines the time steps in the future to look at. Control horizon specifies the number of control action to predict; over this horizon control actions are taken as constants. The optimization problem tries to minimize a cost function using quadratic equations through quadratic programming (QP). This cost function weights both the control actions and the tracking error according to the values given. After the optimal value is calculated, the first control action is applied, and subsequent control actions are discarded and recalculated at the next instant. Constrains are applied to ensure that the calculated control actions do not exceed the physical limits of the system. The system model used to make predictions can be expressed in different forms such as differential equations, state spaces, transfer functions, discrete difference equations. or derived from ANNs.

The cost function used in the MPC is shown in Equation (3), where $J_{y}$ is the cost function of the reference tracking error (4) and $J_{\varepsilon }$ is the cost function of the constraints penalties (5). The cost function can be complemented; for example, it can be added a term to penalize high control output changes between time steps. However, in this research the authors did not implement such terms to prioritize reference tracking as the focus element:

(3) $$J\left(z_{k}\right)=J_{y}\left(z_{k}\right)+J_{\varepsilon }\left(z_{k}\right)$$

Equation (4) expresses the tracking error cost function as the sum of the square of the differences between the reference and the predicted output weighted for all the steps from the instant to the prediction horizon. The importance of this error in the cost function increases as a function of the assigned weight, so that the higher the weight, the more aggressive controllers are obtained, at the cost of greater control efforts:

(4) $$J_{y}\left(z_{k}\right)=\sum_{i=1}^{p}{\left\{w_{i}\left[r(k+i|k)-y(k+i|k)\right]\right\}}^{2}$$

*p* refers to the prediction horizon, $w_{i}$ is the weight of the tracking error, r(k+i|k) is the reference in i steps in the future, and y(k+i|k) is the analogue in the future prediction.

Equation (5) enunciate the cost function of the constrain limit. In practice, constraint violations might be unavoidable. Soft constraints allow a feasible QP solution under such conditions. This cost function depends of the constraint violation penalty weight:

(5) $$J_{\varepsilon }\left(z_{k}\right)=\rho_{\varepsilon }\varepsilon_{k}^{2}$$

where $\rho_{\varepsilon }$ is the constraint violation penalty weight and $\varepsilon_{k}$ the slack variable at control interval *k*.

**Figure 6:**
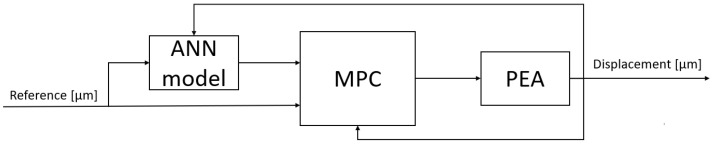
Model Predictive Control (MPC) control scheme.

As shown in Figure 6, in the proposed control scheme, the MPC uses the plant created by the ANN as a model to optimize the cost function and send a control signal to the real PEA plant. The network is fed by the reference and the displacement derivative is provided by feedback from the PEA plant. Due to this internal model in the controller, the MPC sends the optimal control signal to follow the reference provided.

The MPC has been adequately tuned in order to obtain the optimal design parameters. These parameters are shown in Table 2, where the restriction of the input voltage can be highlighted. You would expect the controller to give a signal from 0 to 150 V but due to the hardware used, where the dSPACE board only supports 0–10 V, the controller output must be limited to the range imposed by the board. It is also through hardware, specifically the Thorlabs KPZ101 driver, that the controller 0–10 V signal is transformed into a 0–150 V signal that feeds the PEA. Finally, a sampling time for the control signal of 0.001 s has been chosen. Although a higher sample time would have a lower computational cost, the performance realized by a controller has been found to be lower.

### 4.2. PID

**Figure 7:**
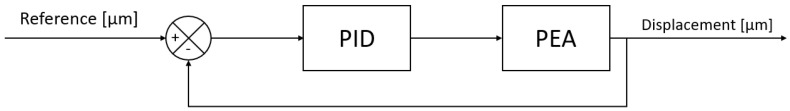
Simple proportional-integral-derivative (PID) on the feedback control scheme.

In order to compare the MPC controller’s performance, a simple and classic approach based on feedback structure is used with a PID controller (Figure 7). This controller is known in advance to perform worse than the MPC, due to the lack of predictability that the PID has. However, the PID is tuned as best as possible using the PID tuner app from MATLAB in order to achieve the best performance (the PID parameters are shown in Table 3).

For the experiment, the standard PID Equation (6) is integrated into the SIMULINK canvas.

(6) $$u\left(k\right)=K_{p}e\left(k\right)+k_{i}\sum_{n=1}^{k}e\left(i\right)\Delta t+K_{d}\left[e\left(k\right)-e\left(k-1\right)\right]$$

## 5. Results

### 5.1. NN Training Results

The training of the ANN was undertaken with data recorded form the sine/triangle input signal described in Section 2.2, which has an amplitude of 150 V and 10 s of a period. As mentioned above in Section 3, the acquired data for training is made up of the input voltage, the displacement and the displacement derivative. A record of 20 s of data was handled and divided in 70/20/10 proportions for training, evaluation, and testing; further details are specified in Table 4. In regards to the hardware used for iteration, a cutting-edge work station, model Dell Precision 3640, was employed with a 0.001 s sample time and configured with parallel calculation activated in 7 cores.

**Figure 8:**
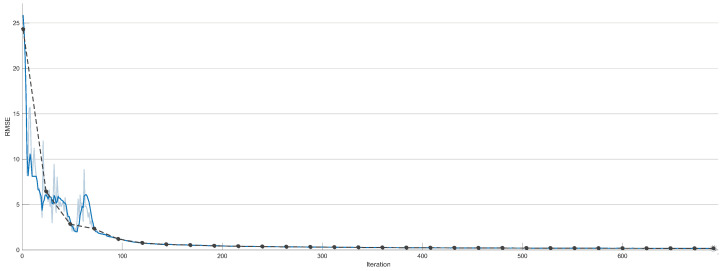
Neural Network training process RMSE.

**Figure 9:**
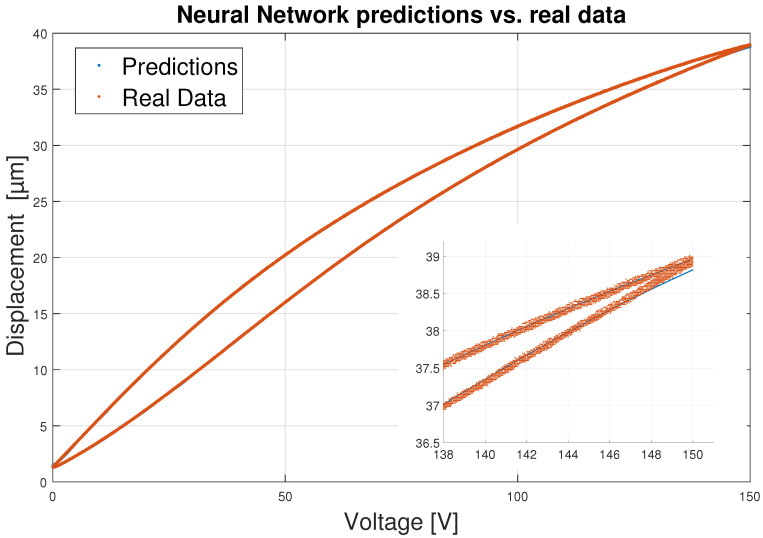
Neural Network predictions vs. real data.

**Figure 10:**
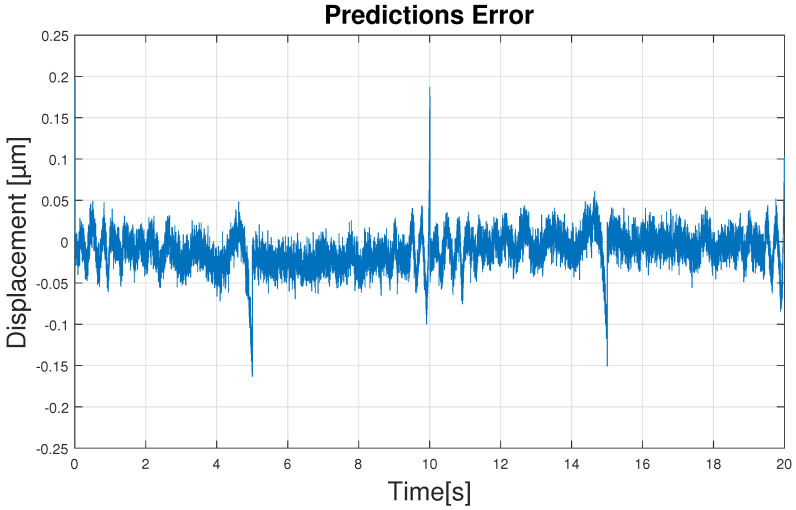
Error of predictions.

Figure 8 shows 700 of the 6000 iterations that have been used to train the network, in order to better observe the initial iterations. In it, it can be seen that the RMSE value drops drastically in the first 150 iterations and then slowly decreases over the following iterations. Due to this fact, it may seem that the network does not need 6000 iterations to be trained, but the network slowly keeps improving and predicting better. After all the training the RMSE value remains at 0.0289—an acceptable result. This fact is reflected in the predictions done by the ANN where the results show an optimal hysteresis model compared to the real PEA data, as shown in Figure 9. Worse predictions are observed at the extremes of hysteresis (at the change of displacement direction), near the maximum displacement value of 38 μm as shown in Figure 9. Nevertheless, these errors have absolute values around 0.15 μm as seen in Figure 10—an acceptable error. Leaving these two areas aside, the error remains constant throughout the hysteresis, fluctuating between 0 and 0.05 μm.

Moreover, the maximum error in all predictions is 0.178 μm and in total a percentage error of 0.158% is obtained. These result can be compared with the ones obtained by the authors of Ref. [57], who obtained maximum modeling errors of 0.35 μm for a NARX and 0.24 micro for a LSTM-NN network.

### 5.2. Reference Tracking Results

The two control structures presented have been embedded in the dSPACE hardware described in Section 2.1, where the experiments have been performed. The signals chosen for the reference are a triangular signal with the maximum possible amplitude (maximum PEA displacement), an identical signal at 1 Hz with a smaller amplitude, a sinusoidal signal at 0.1 Hz and a 15 μm amplitude step signal. In this way, it is be possible to observe the performance of the controllers in different situations. In addition, due to the change of frequency in the signals, the validity of the ANN for a different frequency can be observed. It should be noted that the signal with the possible maximum amplitude goes from 1.4 to 38 μm, instead of from 0 to 38 μm. This is because after the first cycle the PEA displacement does not go below 1.4 μm as explained in Section 2.2.

In this section, the two controller tracking performances are contrasted, indicating the references and the displacement achieved with the two controllers. In addition, the errors produced in the two cases are compared in all experiments. For a better comparison, error indicators are used as shown in Table 5 below. On the one hand, it is proposed to use the Integral Absolute Error (IAE) and Mean Integral Absolute Error (MIAE), which will indicate the quality of the control in absolute and relative terms, respectively. On the other hand, the Root Mean Square Error (RMSE) and Relative Root Mean Square Error (RRMSE) indicators are used to further compare the control results. In Table 5, the terms $e_{i}$ and $r_{i}$ correspond to the error and the reference in sample *i* and datasamples indicates the amount of samples used for the calculation of IAE.

**Figure 11:**
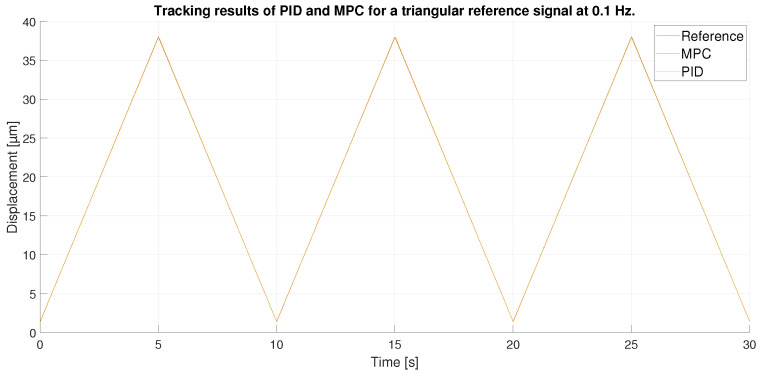
Tracking results of PID and MPC for a triangular reference signal at 0.1 Hz.

**Figure 12:**
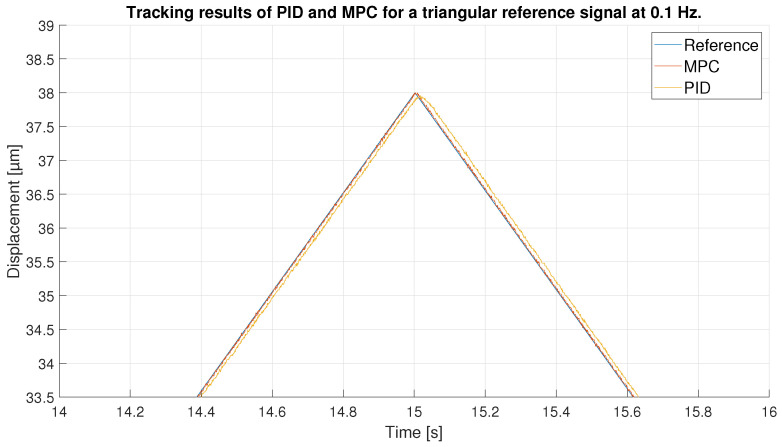
Zoom of tracking results in 0.1 Hz triangular signal peak.

For the triangular signal with maximum amplitude, as shown in Figure 11, the two controllers perform acceptable tracking. However, if we enlarge the image, as in Figure 12, we can see the differences between the two controllers: The MPC shows a higher performance than the PID. The zoom, moreover, is made in the area of greatest error, in the peaks of the triangular signal, where the abrupt change in the reference occurs and therefore, where it is more difficult to perform a precise control. Thus, the performance of the PID in that section is even worse than when the reference is in its straight section. However, the MPC, due to its predictive capability, keeps its tracking capability unchanged throughout the experiment.

**Figure 13:**
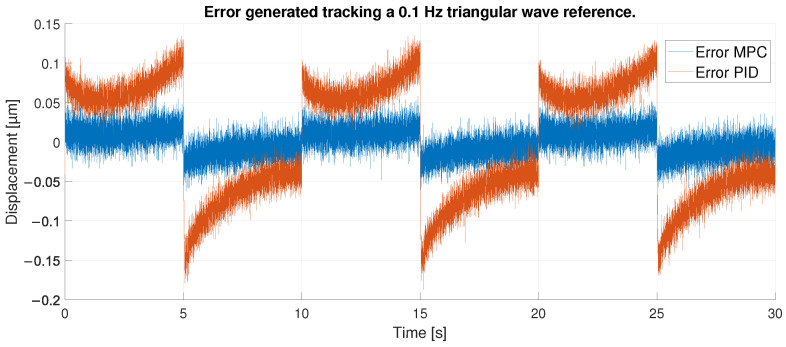
Error of tracking of the PID and MPC for a 0.1 Hz triangular reference.

All these facts can be confirmed by observing Figure 13, where the errors produced in each type of control are compared. As mentioned above, for the PID case the maximum errors occur at the extremes of the hysteresis (maximum and minimum displacement), which due to the frequency of the reference signal occur every 5 s. These have values around 0.17 μm for the upper peaks and around 0.1 μm for the lower peaks, all in absolute terms. therefore, the error in the upper peaks is bigger than in the lower peaks. In addition, at these points of maximum error, due to the change in slope, aggressive behavior is produced where the error reverses its sign into a similar absolute value.

In the case of the MPC, as mentioned above, the error remains constant at less than 1 μm in absolute terms. In the peaks, the change of sign in the error can also be observed, but there is no difference between the upper and lower peaks. After observing the performance of the two controllers it can be concluded that the MPC performs better control compared to the PID, being able to keep the error constant even with abrupt displacement changes.

**Figure 14:**
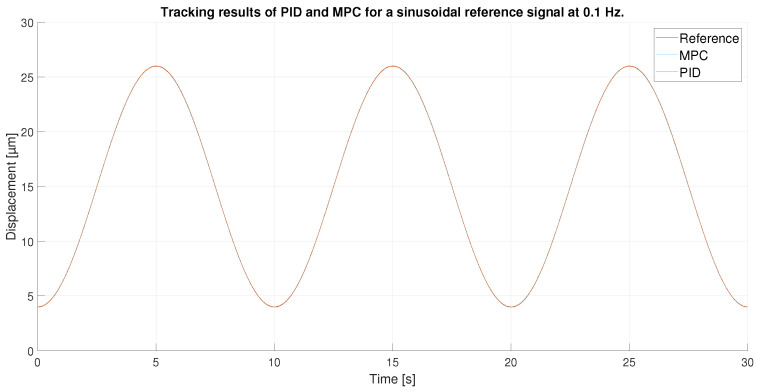
Tracking results of PID and MPC for a sinusoidal reference signal at 0.1 Hz.

For a 0.1 Hz sinusoidal signal as reference, the amplitude is reduced to avoid the peaks of the hysteresis where the control is more difficult to achieve. Therefore, the signal goes from 4 to 26 μm, as shown in Figure 14.

**Figure 15:**
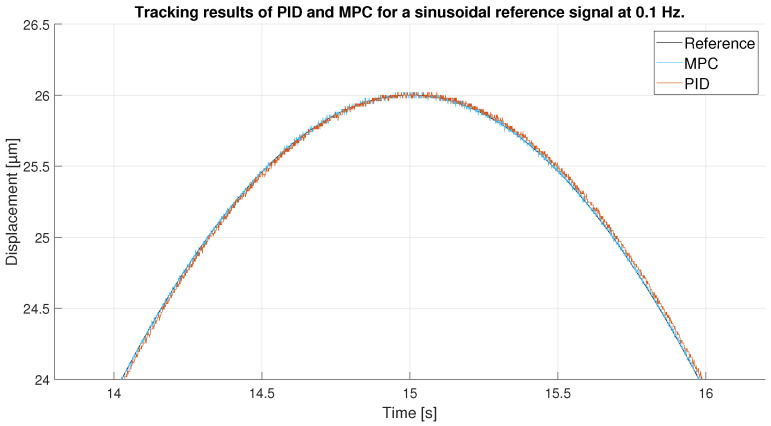
Zoom of tracking results of PID and MPC for a 0.1 Hz sinusoidal reference.

As in the previous experiment, the tracking of the reference signal is generally very good for both controllers, as shown in Figure 14. However, the result in this experiment is even better: because the maximum values of the signal do not reach the hysteresis peaks, the control at these points by the MPC and the PID are good. Furthermore, because the signal is sinusoidal and does not show abrupt changes in directional changes, the control is further improved. Figure 15 shows a zoom in of the previous Figure 14, where the difference between the performance of the MPC and the PID can be seen. It is shown that the the two controllers track well the reference in the extreme of the sinusoidal wave, although the PID shows a worst performance in the ups and downs of the sinusoidal wave.

**Figure 16:**
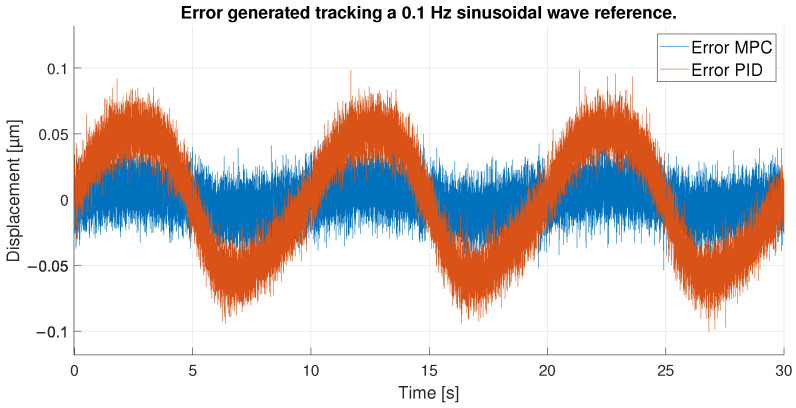
Error of tracking of the PID and MPC for a 0.1 Hz sinusoidal reference.

All these facts can be checked by looking at Figure 16, which shows the tracking error obtained by each of the controllers. The MPC does an exceptional work of keeping its error constant at all times below 0.05 μm. The PID on the other hand, as expected, shows a larger error that fluctuates beyond 0.05 μm. It is interesting to note that in this case, the PID shows the smallest errors when the signal approaches its maximum values as opposed to the previous experiment. This is because in the extremes of the sinusoidal wave the changes in displacement are slower and the PID has time to reduce the tracking error. In the ups and downs of the sinusoidal wave, however, the derivative is bigger and the PID is not able to follow the signal correctly.

For the next experiment, a triangular signal with twice the frequency and lower amplitude was used. The signal ranges from 1.4 to 20 μm at a frequency of 0.2 Hz. Thus, the signal does not reach the high values of the hysteresis curve. Therefore, it is expected to perform better at the high values of this new reference signal than in the first experiment. In addition, the chosen frequency is not the one used to train the ANN, and the MPC has been tuned according to the ANN model, so the results may be worse.

**Figure 17:**
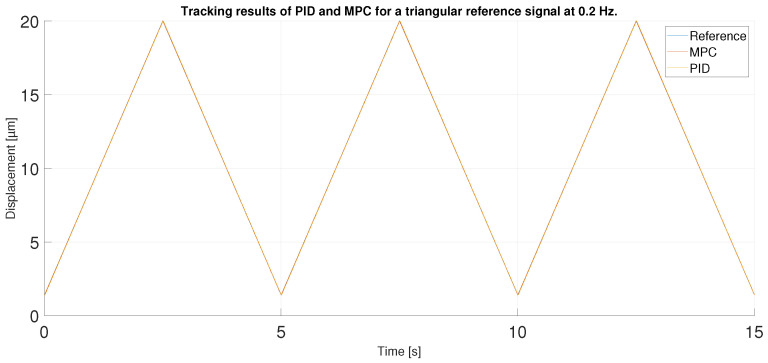
Tracking results of PID and MPC for a triangular reference signal at 0.2 Hz.

**Figure 18:**
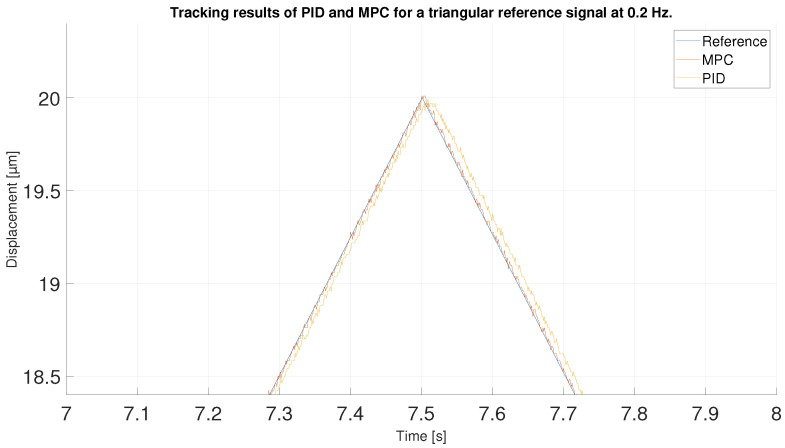
Zoom of tracking results in 0.2 Hz triangular signal peak.

Figure 17 shows the tracking performance of the MPC and PID when following the described triangular reference. Overall, the two controllers again show acceptable performance. To observe in detail the tracking results in the maximum values of the signals it has to be observed the Figure 18 where the MPC shows again a better performance against the PID due to its prediction capabilities.

**Figure 19:**
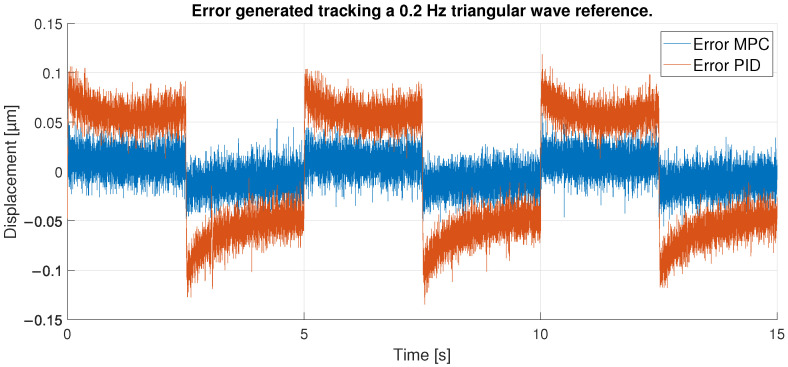
Error of tracking of the PID and MPC for a 0.2 Hz triangular reference.

However, it is in Figure 19 where the differences between the two controllers can really be seen. The MPC shows an error very similar to that seen in the first experiment with the 0.1 Hz triangular signal. This is because the MPC is not affected much by the high values of the hysteresis curve, so even though the signal is easier to control, the MPC shows almost identical performance. The PID, otherwise, is affected by the amplitude change in the signal: compared to the first experiment, the error displayed by the PID is lower at the high values of the reference signal, because it does not approach the high values of the hysteresis curve. In second 5, on the other hand, when the displacement is at the lower peak of the high values of the hysteresis, the error is very similar to that seen in experiment 1.

**Figure 20:**
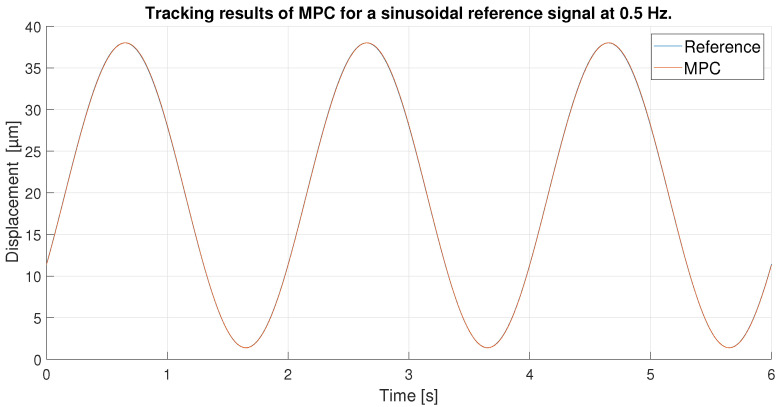
Tracking results of the MPC for a 0.5 Hz sinusoidal reference.

**Figure 21:**
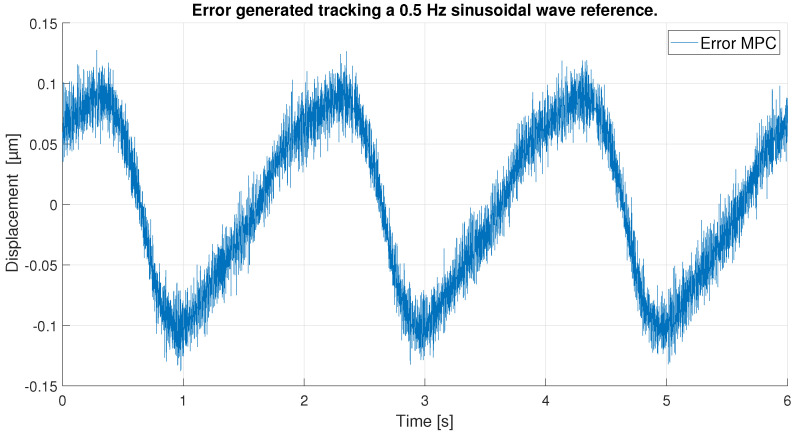
Error of tracking of the MPC for a 0.5 Hz sinusoidal reference.

**Figure 22:**
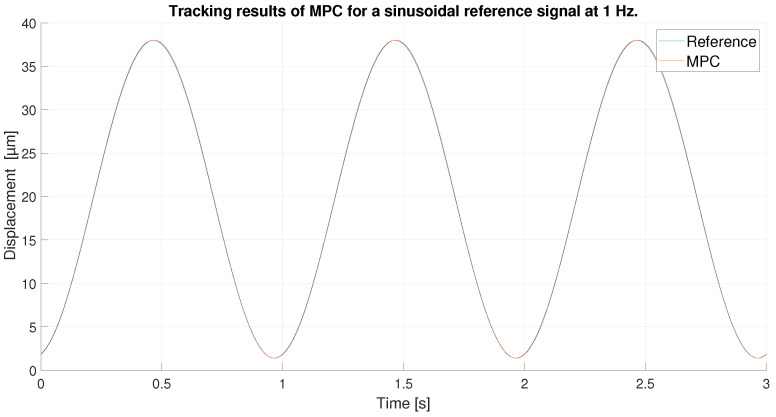
Tracking results of the MPC for a 1 Hz sinusoidal reference.

**Figure 23:**
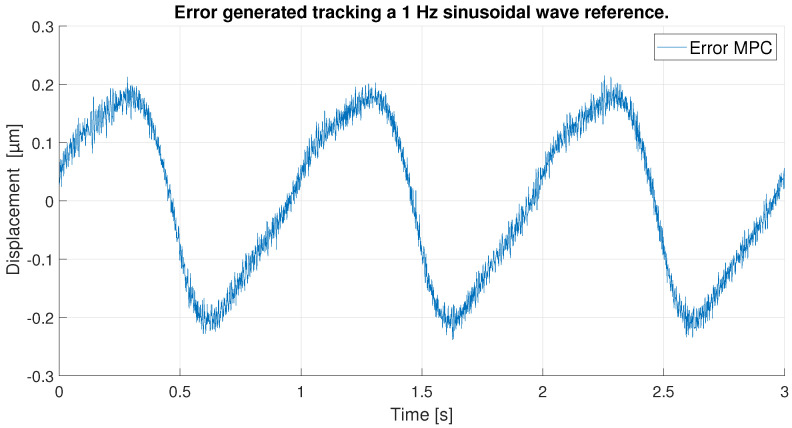
Error of tracking of the MPC for a 1 Hz sinusoidal reference.

**Figure 24:**
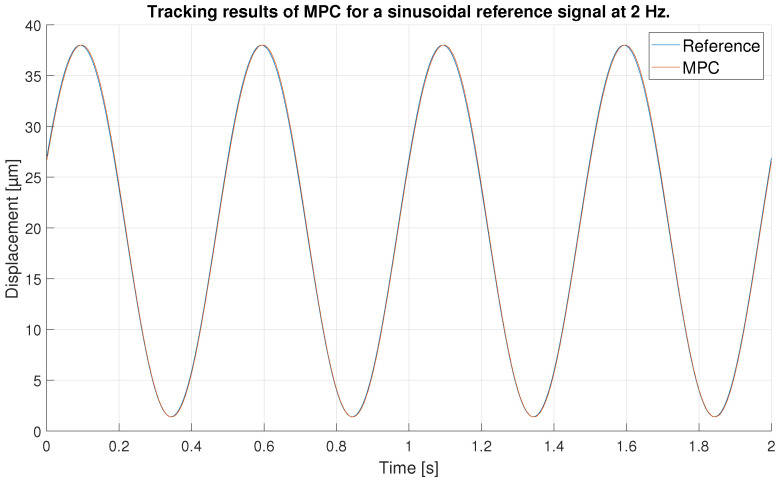
Tracking results of the MPC for a 2 Hz sinusoidal reference of full amplitude.

**Figure 25:**
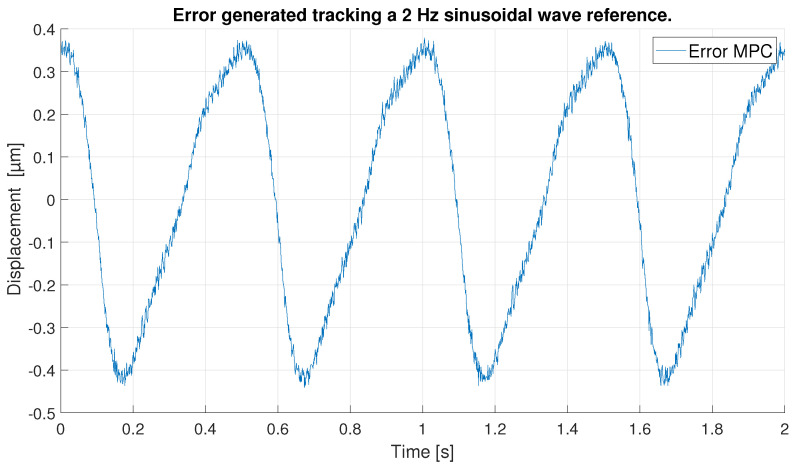
Error of tracking of the MPC for a 2 Hz sinusoidal reference of full amplitude.

**Figure 26:**
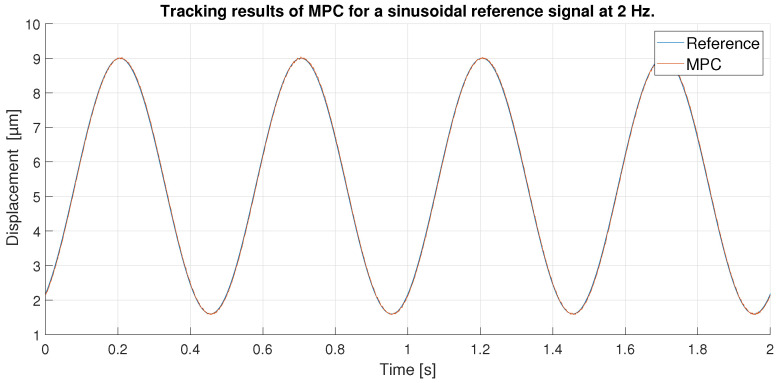
Tracking results of the MPC for a 2 Hz sinusoidal reference of reduced amplitude.

**Figure 27:**
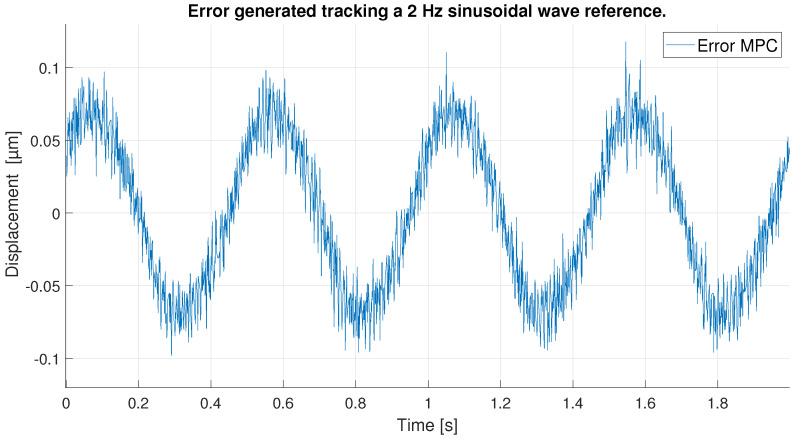
Error of tracking of the MPC for a 2 Hz sinusoidal reference of reduced amplitude.

With respect to the signal frequency, the difference in signal period is not observed to significantly affect the performance of the controllers. This fact indicates that for a reference signal with a frequency around the frequency chosen for the ANN training input, the designed controllers operate correctly. It is true that it cannot be known what frequency range is feasible to maintain the performance of the tuned controllers at 0.1 Hz, because this fact depends on the hysteresis variation versus the frequency variation of each PEA. However, it should be noted that the proposed control scheme can be used for other frequencies and hysteresis because, as it has been pointed out in the experimental validation, if the frequency does not change too much (from 0.1 Hz to 0.2 Hz as discussed in the experiments) the results are good, but for big changes in the frequency, the control performance decays.In order to show this fact and to analyze the robustness of the system, some tests have been carried out at different frequencies, further away from the frequency at which the ANN was trained. Table 6 shows the maximum error and RMSE of the tracking of a sinusoidal function with the maximum amplitude of the PEA for frequencies of 0.5 Hz (Figure 20 and Figure 21), 1 Hz (Figure 22 and Figure 23) and 2 Hz (Figure 24 and Figure 25). These results show, as expected, that the further we move away from the training frequency, the worse results are. Both the maximum error and the RMSE were analyzed. This is due to the fact that the shape of the hysteresis differs more from the reference used as training frequency. Therefore, the approximation made by the network is less accurate. In this sense, if the frequencies are too far, and especially for the higher frequencies, the neural network would have to be trained again with the new data to achieve an accurate model for those frequencies. Tests also demonstrate that with a reference signal of smaller amplitude, the tracking error of the reference is reduced, as shown in Figure 26 and Figure 27 for the 2 Hz case with full and reduced amplitude of 9 μm.

For the last experiment, a step reference signal was used. This step goes from 15 to 30 μm so it has a 15 μm of amplitude. This type of reference can show the performance of the controllers for an instantaneous change of reference with high values, the most difficult type of reference with which to achieve good performance of controllers.

**Figure 28:**
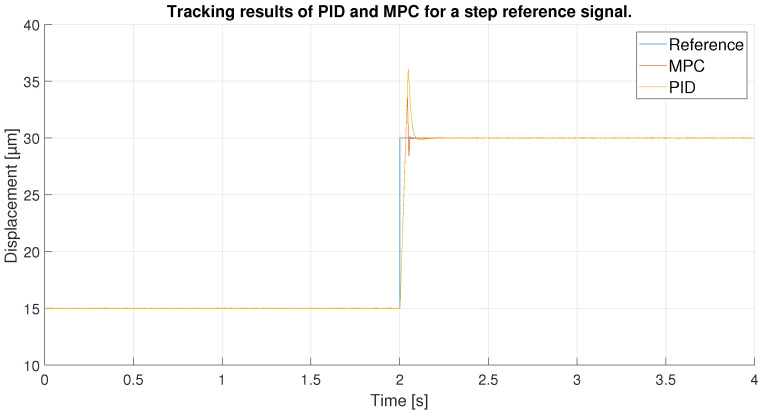
Tracking results of PID and MPC for a step reference signal.

**Figure 29:**
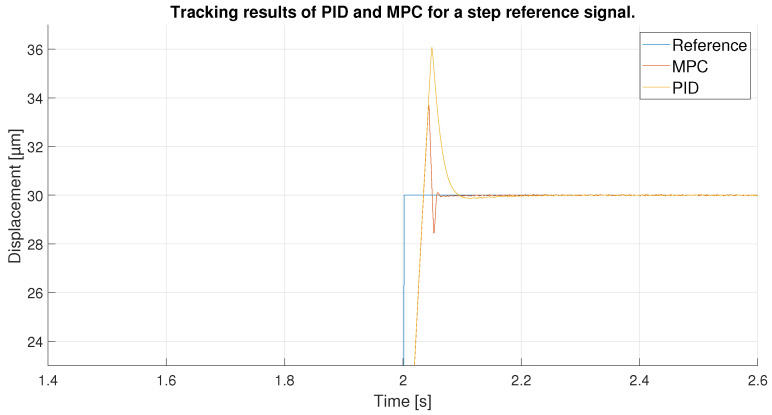
Zoom in of the tracking results in the reference change.

In Figure 28 and the zoom in seen in Figure 29, the result of the tracking of the two controllers is shown. Up to the arrival of the step the tracking is obviously perfect, but as soon as the reference change occurs the controllers try to adapt quickly but do not manage to reach the reference until 0.02 s later. After reaching the reference, the controllers start to differentiate: both show an overshoot, which in the case of the PID is worse as it reaches 36 μm compared to 33.6 μm for the MPC. However, the MPC, when trying to track the reference again, shows an undershoot, unlike the PID, which, although it takes longer, shows a much smaller undershoot. For this reason, the MPC, which behaves more aggressively, manages to stabilize the signal at the reference before the PID.

**Figure 30:**
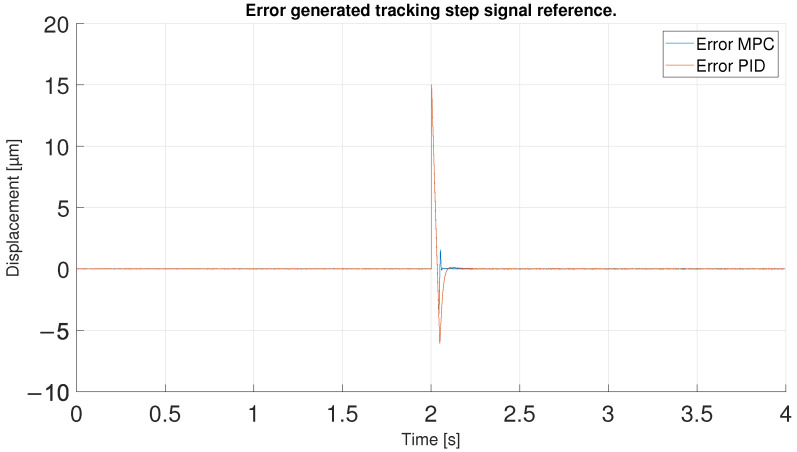
Error of tracking of the PID and MPC for a step reference.

**Figure 31:**
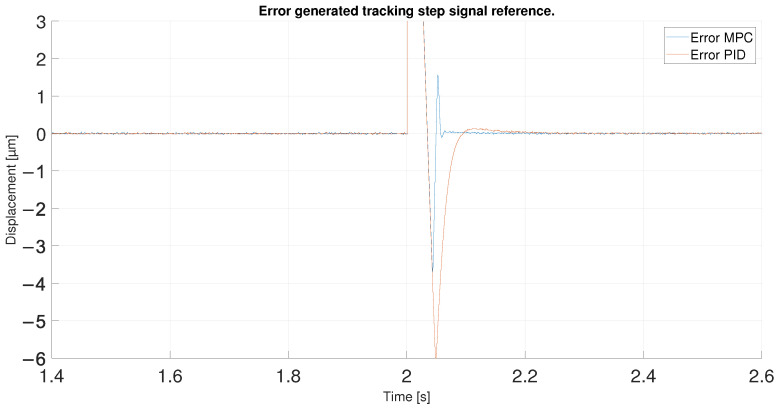
Zoom of error of tracking of the PID and MPC for a step reference.

In order to observe the differences between the two controllers, the tracking errors of the two controllers have been compared in Figure 30. As shown in the previous figures, the error in the reference change is the same for both until the desired value is exceeded, showing an initial error equal to the amplitude of the step signal. After overshooting the reference the first time, the MPC shows errors of 3.7 μm in absolute values compared to 7 μm for the PID. Later, the MPC, with its aggressive control, quickly returns to the reference, again generating an undershoot, reaching an error of 1.5 μm. Even so, the error obtained is lower than the one shown by the PID at that moment because its initial error is higher and it takes longer to return to the reference. Furthermore, as shown in Figure 31, the MPC takes 0.1 s less to stabilize on the reference signal. It should be noted that it could be due to the reduced overshoots in the response of both controllers; however, they would be slower in response.

As mentioned at the beginning of this section, indicators are used to numerically obtain the control results achieved, so that the information obtained from the previous Figures can be contrasted and the two controllers can be better compared. The following Table 7 shows all the indicators values for all the signals and for each controller. It should be noted that these are error indicators; so, the lower the value, the better the tracking obtained by the controller.

Overall, it can be concluded, based on the percentages in the Difference column, which indicate the percentage improvement of a controller with respect to the controller with the worst performance, that the designed MPC greatly outperforms the PID controller: all indicators show a difference of at least 187% (with the exception of the case of the step discussed below), even reaching an improvement of 374%. This fact coincides with the results obtained from the previous Figures, where the MPC shows a better performance in all the experiments. It also coincides with the signals in which the maximum and minimum percentage values have been obtained. The RMSE for the sinusoidal signal shows the lowest improvement of the MPC with 187%, because the sine does not reach the peaks of the hysteresis due to its reduced amplitude and also does not show the abrupt changes that are found in a triangular signal. Therefore the PID can perform better control, approaching the performance of the MPC. On the other hand, when the signal is triangular and the amplitude is the maximum, as is the case with the 0.1 Hz triangular signal, the MPC far outperforms the PID, as shown in the IAE indicator for the 0.1 Hz triangular signal, which shows an improvement of 374%. For the case of step as a reference, the values stand out compared to the other references. The improvements obtained in all the indicators by the MPC with respect to the PID are not so remarkable, reaching only 28% of improvement. This is because the step signal is a single instantaneous change to which the controllers adapt quickly, having the rest of the time a static reference. In this way, both controllers present a similar performance in the steady state.

The indicators in the table not only serve to compare the performance of two controllers, but also to compare the same controller for different reference signals. In the case of the MPC, if the RMSE indicator is observed, it is concluded that the worst performance is for the 0.1 Hz triangular signal, followed by the 0.2 Hz triangular signal and culminating with the best performance for the sinusoidal signal. As mentioned above, the triangular signals show abrupt changes in the peaks, which added to the maximum amplitude for the 0.1 Hz triangular signal, makes control difficult. For the 0.2 Hz triangular signal, the control is better since the amplitude does not reach the upper end of the hysteresis curve so the MPC can perform better tracking. The sinusoidal signal, finally, neither has the maximum possible amplitude nor shows sharp changes, so the MPC can obtain the best performance.

## 6. Conclusions

Piezoelectric actuators have nonlinear effects such as hysteresis, creep, and a rate dependent dynamics, which are challenging problems for achieving an accurate displacement tracking control. Among other non-linearities, hysteresis is the key characteristic that need to be modeled. This article presents the use of ANNs, based on a NARX model, aimed at modeling the hysteresis dynamics of PEAs. This approach moves away from complex mathematical models for modeling hysteresis, yielding a very accurate model. This model has been compared experimentally against a commercial PEA and the prediction errors were limited to 0.5 μm. This ANN, trained to model the hysteresis behavior at 0.1 Hz, served as a model for an MPC controller, due to its good performance to build nonlinear models.

Once the MPC is designed and tuned, it was implemented over the dSPACE control platform over a commercial PEA, supplied by Thorlabs, to experimentally analyze the performance of the control scheme. Different reference signals were used to test the performance of the controller. The obtained results were compared against a PID controller, which is the most common control policy used. The experiments carried out showed that the MPC control scheme obtains better performance at tracking operations.

Experiments show that, even at different reference signals, the MPC controller always obtains better control results and can be up to 370% better when using performance indicators such as IAE. It has also been concluded that the trained neural network, created for frequencies of 0.1 Hz, may be used as a model for higher frequencies, up to at least twice the frequency. However, the ANN would provide lower performance at higher frequencies, and should be retrained for the new desired frequencies.

For future experiments, we intend to compare the performance of the presented approach with other control schemes. In addition, the exact limits of frequencies that the neural network has before being an inaccurate model will be observed. From this, it could be considered to create a frequency-independent network that can model the hysteresis of the PEAs at any frequency. However, it should be noted that the new ANN will require a higher computational cost.

## Figures and Tables

**Table 1:** Thorlabs hardware technical details.

PEA PK4FYC2	Values	Units
Dimensions	7.3 × 7.3 × 36	mm
Impedance at Resonant Frequency	7.3 × 7.3 × 36	mm
Capacitance	7.3 × 7.3 × 36	mm
Operating Temperature	7.3 × 7.3 × 36	mm
Maximum displacement	38.5	μm
Blocking force	1000	N
Resonant frequency	34	kHz
Maximum error	15	%
**Driver Cube KPZ101**		
Output driving voltage for PEA	150	V
Input driving voltage	0–10	V
Maximum output bandwidth	1	kHz
**Reader Cube KSG101**		
Output range	0–10	V
Resolution	1	ηm
**Pre-Amplifier AMP002**		
Output range	0–2	V

**Table 2:** MPC controller’s design parameters.

Parameters	Values	Units
Sample Time	0.001	s
Prediction Horizon	6	time steps
Control Horizon	1	time steps
Output restriction	0–10	V

**Table 3:** Design parameters for the PID.

Constant	Value
$K_{p}$	0.0515
$K_{i}$	27.9564
$K_{d}$	0

**Table 4:** ANN specifications.

Parameters	Values
Data Points	20.000
Training/Validation/Test	70/20/10%
Iterations	6000
Epochs	400
Mini Batch Size	1300 data points
Initial Learn Rate	0.01
Validation frequency	24 iterations
Solver	sgdm
Gradient Threshold Method	absolute-value

**Table 5:** Tracking performance indicators’ equations.

Indicator	Equation
Integral Absolute Error	$IAE=\sum_{i=1}^{N}\left|e_{i}\right|\Delta t$
Mean Integral Absolute Error	MIAE=IAE/datasamples
Root Mean Aquare Error	$RMSE=\sqrt[{}]{1/N\sum_{i=1}^{N}{\left(e_{i}\right)}^{2}}$
Relative Root Mean Square error	$RRMSE=\sqrt[{}]{\sum_{i=1}^{N}{\left(e_{i}\right)}^{2}/\sum_{i=1}^{N}\left(r_{i}\right)}100\%$

**Table 6:** Tracking error for sinusoidal reference far from trained frecuency.

Reference	Maximum Error (μm)	RMSE
Sinusoidal 0.5 Hz	0.1277	0.0888
Sinusoidal 1 Hz	0.2146	0.1327
Sinusoidal 2 Hz	0.3872	0.2638
Sinusoidal 2 Hz (9 μm amplitude)	0.1177	0.0512

**Table 7:** Error comparison between MPC and PID with different source signals.

Reference	IAE		
	MPC	PID	Difference
Triangular 0.1 Hz	0.4151	1.9710	374.83%
Sinusoidal 0.1 Hz	0.4220	1.3546	221.00%
Triangular 0.2 Hz	0.2634	1.1567	339.14%
Step	0.3343	0.4290	28.328%
**Reference**	**IAE MEAN**		
	**MPC**	**PID**	**Difference**
Triangular 0.1 Hz	1.4847 × $10^{-5}$	7.0218 × $10^{-5}$	372.94%
Sinusoidal 0.1 Hz	1.1682 × $10^{-5}$	3.7308 × $10^{-5}$	219.36%
Triangular 0.2 Hz	1.3546 × $10^{-5}$	6.0525 × $10^{-5}$	346.81%
Step	8.3727 × $10^{-5}$	1.0743 × $10^{-4}$	28.3099%
**Reference**	**RMSE**		
	**MPC**	**PID**	**Difference**
Triangular 0.1 Hz	0.0179	0.0731	308.40%
Sinusoidal 0.1 Hz	0.0147	0.0422	187.07%
Triangular 0.2 Hz	0.0167	0.0624	273.65%
Step	0.8248	0.8812	6.8381%
**Reference**	**RRMSE**		
	**MPC**	**PID**	**Difference**
Triangular 0.1 Hz	0.4009	1.6500	311.56%
Sinusoidal 0.1 Hz	0.3692	1.0778	191.92%
Triangular 0.2 Hz	0.5073	1.8869	271.94%
Step	17.3952	18.5835	6.83%

## Data Availability

Not applicable.
